# Immune-Related Gene Expression in Ducks Infected With Waterfowl-Origin H5N6 Highly Pathogenic Avian Influenza Viruses

**DOI:** 10.3389/fmicb.2019.01782

**Published:** 2019-08-02

**Authors:** Siyu Wu, Junsheng Zhang, Jianni Huang, Weiqiang Li, Zhiting Liu, Zhuoliang He, Zuxian Chen, Wanting He, Bingbing Zhao, Zhifeng Qin, Peirong Jiao, Ming Liao

**Affiliations:** ^1^College of Veterinary Medicine, South China Agricultural University, Guangzhou, China; ^2^Shenzhen Academy of Inspection and Quarantine, Shenzhen, China

**Keywords:** duck, immune-related genes, H5N6 avian influenza virus, pathogenicity, transmission

## Abstract

Clade 2.3.4.4 H5 avian influenza viruses (AIVs) are widely prevalent and of significant concern to the poultry industry and public health in China. Nowadays, the clade 2.3.4.4 H5N6 virus has become a dominant AIV subtype among domestic ducks in southern China. We found that waterfowl-origin clade 2.3.4.4 H5N6 viruses (A/goose/Guangdong/16568/2016, GS16568 and A/duck/Guangdong/16873/2016, DK16873) isolated from southern China in 2016 could replicate in multiple organs of inoculated ducks. DK16873 virus caused mild infections and killed 2/5 of inoculated ducks, and GS16568 virus did not kill inoculated ducks. In addition, the two viruses could be transmitted via direct contact between ducks. DK16873 and GS16568 viruses killed 2/5 and 1/5 of contact ducks, respectively. Furthermore, ducks inoculated with the two H5N6 viruses exhibited different expressions of immune-related genes in their lungs. The expression of RIG-I, TLR3 and IL6 was significantly upregulated at 12 h post-inoculation (HPI) and most of the tested immune-related genes were significantly upregulated at 3 days post-inoculation (DPI). Notably, the expression of RIG-I and IL-6 in response to DK16873 virus was significantly higher than for GS16568 virus at 12 HPI and 3 DPI. Our research have provided helpful information about the pathogenicity, transmission and immune-related genes expression in ducks infected with new H5N6 AIVs.

## Introduction

Avian influenza virus (AIV) is a negative-sense RNA virus that belongs to the orthomyxoviridae family (Webster et al., 1992). AIV can be classified into highly pathogenic avian influenza (HPAI) virus and low pathogenic avian influenza (LPAI) virus based on pathogenicity in chickens.

Since the first H5N1 HPAI virus was detected in 1996, these viruses have been prevalent among poultry in Asia, Europe, and Africa. This situation has resulted in heavy losses in the poultry industry (Alexander and Brown, 2009). More importantly, these viruses can cause human infections and are of great concern to public health (Peiris et al., 2007). From June 2003 through February 2019, there have been 860 cases of confirmed human infections with H5N1 viruses, and 20 cases of human infections with H5N6 viruses (WHO, 2019). Since 2008, clade 2.3.4 H5N2 (Zhao et al., 2012), H5N5 (Gu et al., 2011), and H5N8 (Song et al., 2015) viruses have been detected in China. These viruses have continued to evolve into different subclades (e.g., clade 2.3.4.4) (WHO, 2015). Recent studies have shown that the new clade 2.3.4.4 H5N6 virus was dominant in southern China, especially in ducks (Bi et al., 2016). However, the pathogenicity and transmission of ducks infected with H5N6 virus remain unclear.

The virulence of AIV is not only determined by viruses but is also related to host factors (Fukuyama and Kawaoka, 2011; Tscherne and Garcia-Sastre, 2011). The basis of innate immunity is the activation of host immune signaling pathways mediated by pattern recognition receptors (PRRs) (Akira et al., 2006). Reports have demonstrated that RIG-I and MDA5 are involved in the immune response of ducks infected with H5N1 virus (Barber et al., 2010; Wei et al., 2014). Pro-inflammatory and interferon-stimulated genes (ISGs) have also been associated with the host immune response to ducks infected with the H5N1 virus (Saito et al., 2018). However, the expression of immune-related genes in ducks infected with different pathogenicity H5N6 viruses remains unclear.

Here, we systematically studied the pathogenicity, transmission, and expression of immune-related genes in ducks infected with two waterfowl-origin H5N6 AIVs isolated from southern China in 2016.

## Materials and Methods

### Viruses

Clade 2.3.4.4 H5N6 HPAIVs, A/duck/Guangdong/16873/2016 (DK16873) and A/goose/Guangdong/16568/2016 (GS16568) used in this study were isolated from swabs of apparently healthy ducks and geese, which came from different areas, in a live bird market in Guangdong of China in May to August, 2016. These H5N6 viruses were purified and propagated in 9-day-old specific-pathogen-free (SPF) embryonated hen eggs, as described in the literature (Jiao et al., 2018). The values of 50% egg infective doses (EID_50_) were calculated using the Reed-Muench method (Thakur and Fezio, 1981). All of the experiments were conducted in animal biosafety level 3 (ABSL-3) facilities.

### Animal

Four-week-old healthy Muscovy ducks were purchased from farms in Guangdong and housed in the isolators of ABSL-3 facilities. The domestic ducks were confirmed to be serologically negative for avian influenza with a hemagglutination inhibition (HI) test.

### Pathogenicity and Transmission Experiments of H5N6 Virus in Ducks

To determine the pathogenicity of two H5N6 AIVs in ducks, the animals in each inoculated group (14 ducks) were inoculated intranasally with 10^6^ EID_50_ of GS16568, and DK16873 viruses in a 200 uL volume, respectively. Furthermore, 14 ducks were inoculated intranasally with 200 uL phosphate buffered saline (PBS) as a control group. To investigate the transmission of these viruses, five ducks were placed in each inoculated group for physical contact at 24 h post-inoculation (HPI). Each duck was labeled with a number metal ring for identification. At 12 HPI, 3 and 5 days post-inoculation (DPI), three ducks in each inoculated group were euthanized humanely to examine the viral distribution in the liver, spleen, lung, kidney, brain, intestine, trachea, pancreas, and bursa of Fabricius. Three ducks in control group were also euthanized humanely to collect lungs for further use. The remaining ducks were monitored for clinical signs or death until 14 DPI. Oropharyngeal and cloacal swabs from all the ducks were collected at 3, 5, 7, 9, 11 and 13 DPI to detect virus shedding. The surviving ducks were euthanized humanely at 14 DPI to collect serum. Seroconversion of all surviving ducks was confirmed with a HI test. All of the collected tissues and swabs were titrated for virus infectivity in SPF embryonated hen eggs using the EID_50_ method, as described in the literature (Jiao et al., 2018). Virus titers were calculated using the Reed-Muench method (Thakur and Fezio, 1981). All of the animal experiments were conducted in accordance with experimental animal administration guidelines and the guidelines of the ethics committee of South China Agriculture University.

### Quantification of Immune-Related Gene Expression in the Lungs of H5N6 Virus-Inoculated Ducks

To investigate the host immune response of ducks inoculated with the H5N6 AIV, we quantified the expression of immune-related genes (TLR7, TLR3, RIG-I, MDA5, IFN-α, IFN-β, IL-6, IL-8, Mx, and MHC-I) in the lungs of inoculated ducks. Total RNA was extracted from the lungs of inoculated ducks and control ducks at 12 HPI and 3 DPI using an Eastep^®^ Super Total RNA Extraction Kit (Promega, United States) according to the manufacturer’s instructions. Total RNA (1ug) was reverse-transcribed with the M-MLV Reverse Transcriptase (Promega) according to the manufacturer’s protocol. The cDNA was stored at −80°C for further use.

Quantitative real-time (qRT) PCR was conducted using a GoTaq^®^ qPCR Master Mix (Promega). The qRT-PCR primers (Table 1) were designed using Oligo 7 software. Primer pairs were selected according to specificity determined by dissociation curves and nucleotide sequencing. qRTPCR was run on a Bio-Rad CFX96 Touch^TM^ Real-Time PCR Detection System (Bio-Rad Laboratories, United States) using the following cycle parameters: 1 cycle of 95°C for 5 min, followed by 40 cycles of 95°C for 15 s, and 60°C for 34°s. Relative target gene expressions were calculated using the 2^–ΔΔ^ ^Ct^ method and expressed as a fold change in gene expression compared with control ducks (Jiao et al., 2018). GAPDH was used as the reference endogenous gene to normalize the quantification of the target gene.

**TABLE 1:** Primer sequences used in the quantitative real-time PCR.

**Gene**	**Primer sequences (5′ to 3′)**	**Accession No.**
TLR7	F: CCTTTCCCAGAGAGCATTCA	AY940195
	R: TCAAGAAATATCAAGATAATCACATCA	
TLR3	F: GAGTTTCACACAGGATGTTTAC	JQ910167
	R: GTGAGATTTGTTCCTTGCAG	
RIG-I	F: CTGGCAGAAGCAATTGAGAAC	EU363349
	R: TGCTGAATCTCTTCACACTCC	
MDA5	F: CTTGCAGATGATTTAAGTGGA	KJ451070
	R: CTTCACTACAGAATGTCCTGG	
IFN-α	F: TCCTCCAACACCTCTTCGAC	KF731866
	R: GGGCTGTAGGTGTGGTTCTG	
IFN-β	F: CAGCATCAACAAGTACTTCA	KM035791
	R: CTTCCGAAGTGGCTGGGAGA	
Mx	F: CCAGACCTGACACTAATTGAT	KR025554
	R: CACATTACATGGCACCACTAC	
IL6	F: CAGACCTACCTTGAATACGTA	AB191038
	R: AGCTGAATCTGGGATGACCAC	
IL8	F: CCGGTGCCAGTGCATAAGCAC	DQ393274
	R: ATGATTTCAACGTTCTTGCAG	
MHCI	F: GAAGGAAGAGACTTCATTGCCTTGG	AB115246
	R: CTCTCCTCTCCAGTACGTCCTTCC	
GAPDH	F: ATGTTCGTGATGGGTGTGAA	AY436595
	R: CTGTCTTCGTGTGTGGCTGT	

### Statistics and Analysis

**FIGURE 1:**
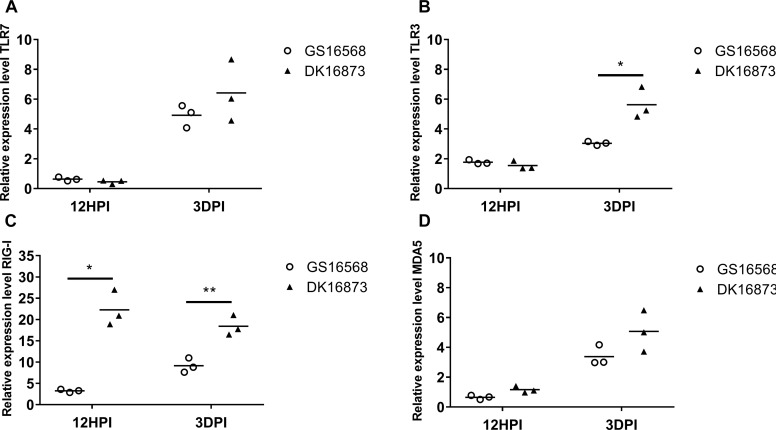
Relative expression of Pattern-recognition receptors (PRRs) in the lung of ducks inoculated with GS16568 and DK16873 viruses. At 12 h post-inoculation (HPI) and 3 days post-inoculation (DPI), qRT-PCR were used to quantified the expression of PRRs in the lungs of H5N6 virus-infected ducks, that were expressed relative to the geometric mean of expression in control ducks. **(A)**TLR7, **(B)** TLR3, **(C)** RIG-I, and **(D)** MDA5. Each dot represents one duck. Each dot represents the level of target gene mRNA relative to mock after normalizing to GAPDH. Statistical analysis was performed using a paired Student’s *t*-test (^*^p < 0.05, ^∗∗^*p* < 0.01).

**FIGURE 2:**
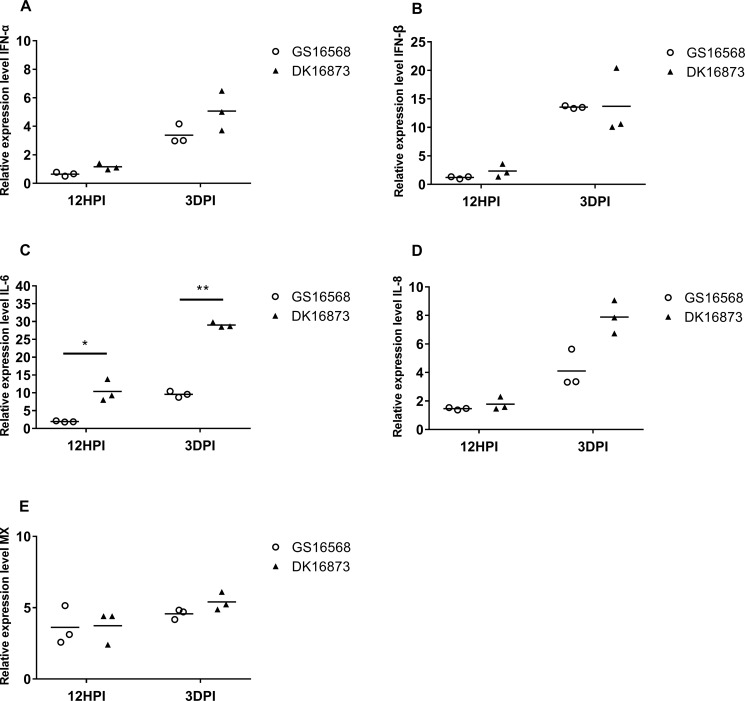
Relative expression of IFNs, proinflammatory cytokines, and ISGs in the lung of ducks inoculated with GS16568 and DK16873 viruses. At 12 HPI and 3 DPI, qRT-PCR were used to quantified the expression of IFNs, proinflammatory cytokines and ISGs in the lungs of H5N6 virus-infected ducks, that were expressed relative to the geometric mean of expression in control ducks. **(A)** IFNα, **(B)** IFN-β, **(C)** IL-6, **(D)** IL-8, and **(E)** Mx. Each dot represents one duck. Each dot represents the level of target gene mRNA relative to mock after normalizing to GAPDH. Statistical analysis was performed using a paired Student’s *t*-test (^*^p < 0.05, ^∗∗^*p* < 0.01).

**FIGURE 3:**
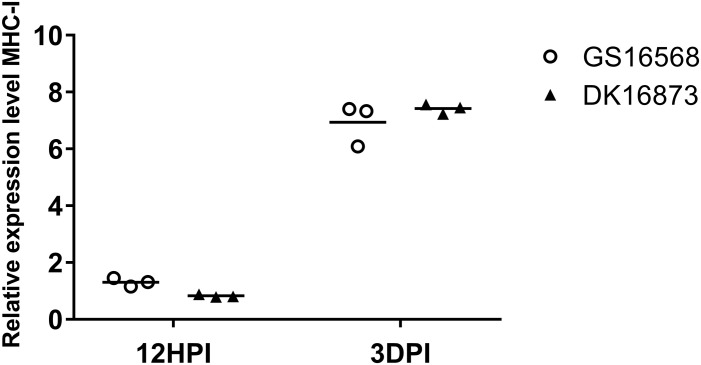
Relative expression of MHC-I in the lung of ducks inoculated with GS16568 and DK16873 viruses. At 12 HPI and 3 DPI, qRT-PCR were used to quantified the expression of MHC-I in the lungs of H5N6 virus-infected ducks, that were expressed relative to the geometric mean of expression in control ducks. Each dot represents one duck. Each dot represents the level of target gene mRNA relative to mock after normalizing to GAPDH. Statistical analysis was performed using a paired Student’s *t*-test (^*^p < 0.05, ^∗∗^*p* < 0.01).

The statistical analyses were conducted using GraphPad Prism 7.0 software (GraphPad Software Inc, United States). The Fisher’s exact test were applied to compare mortality rates between the groups in Table 2. The Student’s t-test was used in Figures 1–3. The *p* < 0.05 and *p* < 0.01 were considered to indicate a statistically significant difference (^*^p < 0.05; ^∗∗^*p* < 0.01).

**TABLE 2:** Illness and mortality of ducks after inoculated with H5N6 influenza viruses.

**Strains**	**Titer(log_10_EID_50_)**	**Group**	**Illness**	**Death**	**MDT**	**Virus replication in dead ducks’ lung(log_10_EID_50_/100 mg)^a^**
GS16568	7.00	Inoculated^b^	0/5	0/5		
		Contact^c^	0/5	1/5	4.0	6.50 ± 0
DK16873	8.83	Inoculated	3/5	2/5	5.5	6.50 ± 0
		Contact	2/5	2/5	5.5	5.88 ± 0.38

*MDT, mean dead time. ^*a*^For statistical analysis, a value of 1.5 was assigned if the virus was not detected from the undiluted sample in three embryonated hen eggs (Jiao et al., 2018). Viral titers were expressed as mean ± SD in log_10_EID_50_/100 mg of tissue. ^*b*^Ducks inoculated with virus. ^*c*^Naive contact ducks housed with those inoculated.*

### Ethics Statements

This study was carried out in ABSL-3 facilities in compliance with approved protocols by the biosafety committee of South China Agriculture University. All animals experiment were handled in accordance with the principles of the Basel Declaration and recommendations of the approved guidelines of the Experimental Animal Administration and Ethics Committee of South China Agriculture University. The protocol (SCAUABSL2017-005) was approved by the experimental Animal Administration and Ethics Committee of the South China Agricultural University.

## Results

### Pathogenicity and Replication of H5N6 in Ducks

To investigate the pathogenicity of the H5N6 HPAIVs in ducks, we inoculated ducks with 10^6^ EID_50_ of A/goose/Guangdong/16568/2016 (GS16568), and A/duck/Guangdong/16873/2016 (DK16873) viruses in a 200 uL volume, respectively. At 12 HPI, 3 and 5 DPI, three ducks in each inoculated group were euthanized humanely to test for viral replication in the liver, spleen, lung, kidney, brain, intestine, trachea, pancreas, and bursa of Fabricius, respectively. All of the ducks were monitored for clinical signs or death for 14 days.

After inoculation with GS16568 virus, none of the ducks died or exhibited obvious clinical symptoms (Table 2). However, of the five DK16873-inoculated ducks that remained after day 5, three ducks exhibited mild clinical symptoms, and two of them died within 6 DPI (Table 2). The Fisher’s exact test showed that the mortality rate of inoculated ducks caused by the two viruses was not significantly different. All of the inoculated ducks seroconverted at 14 DPI, which suggested that all the inoculated ducks were infected with H5N6 HPAIVs.

At 12 HPI, GS16568 virus was detected in 7 of 9 organs in the inoculated ducks: the liver, lung, kidney, intestine, trachea, pancreas, and bursa of Fabricius. Their mean titers were 1.58, 2.00, 1.58, 2.50, 3.50, 2.25, and 1.75 log_10_EID_50_/100 mg, respectively (Table 3). DK16873 virus was detected in 8 of 9 organs in the inoculated ducks: the liver, spleen, lung, kidney, intestine, trachea, pancreas, and bursa of Fabricius. Their mean titers were 2.83, 3.00, 3.83, 2.17, 2.33, 3.58, 2.25, and 2.50 log_10_EID_50_/100 mg, respectively. These data indicated that both the two H5N6 viruses could replicate in ducks at the early stage of viral infection. At 3 DPI, GS16568, and DK16873 virus replicated systematically in all of the detected organs: the liver, spleen, lung, kidney, brain, intestine, trachea, pancreas, and bursa of Fabricius. The mean titers were 5.50–4.58, 6.42–5.58, 5.33–5.58, 6.50–4.50, 4.17–4.50, 5.58–5.42, 5.42–6.17, 3.92–4.33, and 5.83–6.50 log_10_EID_50_/100 mg, respectively. At 5 DPI, GS16568 and DK16873 replicated to mean titers of 4.50–4.42, 5.17–5.83, 3.75–4.08, 4.67–5.42, 2.75–4.42, 3.92–5.50, 4.58–6.17, 2.50–4.92, and 5.08–5.33 log_10_EID_50_/100 mg in the liver, spleen, lung, kidney, brain, intestine, trachea, pancreas, and bursa of Fabricius, respectively (Table 3). Overall, both the two viruses were able to replicate in multiple organs of inoculated ducks.

**TABLE 3:** Replication of H5N6 avian influenza viruses in ducks.

**Strains**	**Time**	**Virus replication in organs (log_10_EID_50_/100 mg)^a^**								
		**Liver**	**Spleen**	**Lung**	**Kidney**	**Brain**	**Intestine**	**Trachea**	**Pancreas**	**Bursa of Fabricius**
GS16568	12 HPI	1.58 ± 0.14	ND^b^	2.00 ± 0.20	1.58 ± 0.14	ND	2.50 ± 1.73	3.50 ± 0.25	2.25 ± 0.87	1.75 ± 0.43
	3 DPI	5.50 ± 1.75	6.42 ± 0.14	5.33 ± 0.88	6.50 ± 0	4.17 ± 0.80	5.58 ± 0.88	5.42 ± 0.76	3.92 ± 1.51	5.83 ± 0.58
	5 DPI	4.50 ± 1.75	5.17 ± 1.28	3.75 ± 2.17	4.67 ± 1.61	2.75 ± 1.39	3.92 ± 1.42	4.58 ± 2.70	2.50 ± 1.52	5.08 ± 1.51
DK16873	12 HPI	2.83 ± 1.18	3.00 ± 1.39	3.83 ± 1.84	2.17 ± 0.63	ND	2.33 ± 1.01	3.58 ± 1.01	2.25 ± 1.09	2.50 ± 0
	3 DPI	4.58 ± 1.01	5.58 ± 1.15	5.58 ± 0.88	4.50 ± 0.25	4.50 ± 0.25	5.42 ± 0.14	6.17 ± 0.58	4.33 ± 1.04	6.50 ± 0
	5 DPI	4.42 ± 1.13	5.83 ± 0.58	4.08 ± 1.04	5.42 ± 0.14	4.42 ± 1.59	5.50 ± 0	6.17 ± 0.38	4.92 ± 0.52	5.33 ± 0.14

*HPI, hour post-inoculation; DPI, day post-inoculation. Ducks were inoculated intranasally with 10^6^ EID_50_ of the GS16568 and DK16873 viruses in a volume of 200 uL; three ducks in each group were randomly chosen for virus titer at 12, 3, and 5 DPI and the lung, liver, spleen, kidney, brain, trachea, pancreas, intestine, and cloacal bursa of the three chosen ducks were collected for virus titer in eggs. ^*a*^For statistical analysis, a value of 1.5 was assigned if the virus was not detected from the undiluted sample in three embryonated hen eggs. Viral titers were expressed as mean ± SD in log_10_EID_50_/100 mg of tissue. ^*b*^Not detected.*

### Shedding of H5N6 Influenza Viruses in Ducks

To evaluate virus shedding in inoculated ducks, we collected oropharyngeal and cloacal swabs on 3, 5, 7, 9, 11, and 13 DPI. The swabs were inoculated into the SPF embryonated hen eggs to detect the virus. Virus shedding was determined based on the results of virus detection.

Shedding of GS16568 virus was detected in both the oropharyngeal and cloacal swabs of inoculated ducks from 3 to 9 DPI (Table 4). Shedding of DK16873 virus was detected in oropharyngeal swabs from 3 to 9 DPI. Shedding of DK16873 virus was detected in cloacal swabs from 3 to 7 DPI (Table 4).

**TABLE 4:** Viral shedding in cloacal and oropharyngeal swabs from inoculated and contacted ducks.

**Strain**	**Infection sample**	**3 DPI**		**5 DPI**		**7 DPI**		**9 DPI**		**11 DPI**		**13 DPI**	
		**T**	**C**	**T**	**C**	**T**	**C**	**T**	**C**	**T**	**C**	**T**	**C**
GS16568	Inoculated^a^	11/11	10/11	7/8	2/8	2/5	2/5	3/5	1/5	0/5	0/5	0/5	0/5
	Contacted^b^	5/5	5/5	3/4	0/4	3/4	3/4	1/4	0/4	0/4	1/4	2/4	0/4
DK16873	Inoculated	11/11	11/11	6/8	6/8	3/3	2/3	1/3	0/3	0/3	0/3	0/3	0/3
	Contacted	5/5	5/5	4/4	4/4	3/3	3/3	3/3	1/3	2/3	2/3	0/3	0/3

*DPI, day post-inoculation; T, oropharyngeal swab; C, cloacal swab. ^*a*^Ducks inoculated with virus. ^*b*^Naive contact ducks housed with those inoculated.*

### Transmission of H5N6 in Ducks

In order to determine the transmission of GS16568 and DK16873 viruses in ducks, five ducks were raised together with each inoculated group at 1 DPI. Both oropharyngeal and cloacal swabs were collected at 3, 5, 7, 9, 11, and 13 DPI to test viral shedding of the naïve-contact ducks.

GS16568-contacted ducks did not exhibit obvious clinical signs during the observation period, and one duck died at 4 DPI (Table 2). Shedding of DK16568 virus was detected in the oropharyngeal swabs of contact ducks at 3, 5, 7, 9, and 13 DPI (Table 4). Shedding of DK16568 virus was detected in the cloacal swabs of contact ducks at 3, 7, and 11 DPI. Of the five DK16873-contacted ducks, two of them exhibited mild depression, and died within 6 DPI. DK16873-contacted ducks shed viruses in both oropharyngeal and cloacal swabs from 3 to 11 DPI. Additional tests revealed that GS16568 and DK16873 viruses were replicated in the tested tissues of all dead contact ducks (Supplementary Table S1). The results of Fisher’s exact test showed that the mortality rate of these contact ducks caused by the two viruses was not significantly different. In summary, both GS16568 and DK16873 viruses were shed from the respiratory tract and the digestive tract of the inoculated ducks and contact ducks, which indicated that the two viruses could be efficiently transmitted via direct contact between ducks.

### Expression of PRRs in the Lungs of H5N6-Infected Ducks

Previous studies have shown that PRRs are involved in host immune response against influenza virus (Chen et al., 2013; Iwasaki and Pillai, 2014). Here, we quantified the mRNA level of TLR7, TLR3, RIG-I and MDA5 in duck lungs at 12 HPI and 3 DPI to assess the expression of PRRs in H5N6 virus-infected ducks.

The mRNA expression of TLR7 was very slightly downregulated at 12 HPI and significantly upregulated at 3 DPI after infected with GS16568 and DK16873 viruses (Figure 1A). The expression of TLR3 was slightly increased at 12 HPI and significantly increased at 3 DPI in response to the two viruses (Figure 1B). The expression of RIG-I was increased after the birds were infected with the two viruses at 12 HPI and 3 DPI (*P* < 0.05 and *p* < 0.01, respectively) (Figure 1C). Notably, the expression of TLR3 and RIG-I in response to DK16873 virus was higher than that of GS16568 virus at 3 DPI (*P* < 0.05 and *P* < 0.01, respectively). The expression of MDA5 was upregulated in response to the two viruses at 3 DPI (Figure 1D). Therefore, the expression of PRRs in response to the two viruses was increased at 3 DPI in the lungs of H5N6 virus-infected ducks.

### Expression of IFNs, Proinflammatory Cytokines, and ISGs in the Lungs of H5N6-Infected Ducks

Studies have demonstrated that interferons (IFNs), antiviral cytokines and proinflammatory cytokines are involved in the immune response of H5N1 virus infection (Cheung et al., 2002). To analyze the expression of these genes in ducks infected with H5N6 viruses of varying pathogenicity, we quantified the mRNA level of IFN-α, IFN-β, Mx, IL-6, and IL-8 in the birds’ lungs at 12 HPI and 3 DPI.

The expression of IFN-α was slightly downregulated, yet IFN-β was slightly upregulated in response to the two viruses at 12 HPI. However, the expression of both IFN-α and IFN-β was increased after the birds were infected with the two viruses at 3 DPI (Figures 1A,B). At 12 HPI and 3 DPI, the expression of IL-6 in DK16873 virus-infected ducks (10.38- fold; *P* < 0.05 and 29.01-fold; *P* < 0.01, respectively) was significantly higher than that of GS16568 virus-infected ducks (1.95-fold and 9.60-fold, respectively; *P* < 0.05) (Figure 2C). The expression of IL-8 was slightly increased at 12 HPI and significantly increased at 3 DPI in response to DK16873 virus (Figure 2D). The expression of Mx was upregulated in response to the two viruses at 12 HPI and 3 DPI, with a fold increase ranging from 3.62 to 5.41 (Figure 2E). Therefore, our results demonstrated that IFN-α, IFN-β, Mx, IL-6 and IL-8 were involved in the immune response of ducks infected with H5N6 HPAIVs.

### Expression of MHC-I in the Lungs of H5N6-Infected Ducks

MHC molecules are responsible for presenting processed antigens and increasing specific immunity via the activation of B and T cells to eliminate viruses (Germain, 1994). To compare the expression of MHC-I in ducks infected with H5N6 viruses of different pathogenicity, we tested the mRNA of MHC-I in the lungs of ducks at 12 HPI and 3 DPI.

At 12 HPI, the expression of MHC-I was slightly increased in response to GS16568 virus infection, yet decreased in response to DK16873 virus infection (Figure 3). At 3 DPI, the expression of MHC-I was increased after the birds were infected with the GS16568 and DK16873 viruses. These results reveal that MHC-I was involved in the duck immune response to H5N6 virus infection.

## Discussion

As a natural host of AIVs, ducks can survive even when they are infected with HPAIVs (Chen et al., 2004). However, since 2003, reports have demonstrated that clade 2.3.4 H5N1 HPAI viruses can cause disease and kill all of infected ducks (Li et al., 2010). Previous studies have shown that clade 2.3.4.4 H5N2, H5N5 and H5N8 HPAI viruses isolated from China from 2010 to 2016 exhibited mild to low virulence in ducks and were not lethal (Zhao et al., 2013; Liu et al., 2018). Other investigations have also demonstrated that clade 2.3.4.4 H5N6 viruses isolated in 2014 in eastern China caused systematic infections in ducks (Sun et al., 2016). Recent studies have demonstrated that clade 2.3.4.4 H5N6 HPAIVs exhibited different pathogenicity in ducks (Uchida et al., 2019). Epidemiological studies have shown that clade 2.3.4.4 H5N6 virus was dominant in waterfowl in southern China from 2014 to 2016 (Bi et al., 2016). However, the pathogenicity and transmission of ducks infected with the new H5N6 HPAIVs isolated from waterfowl in southern China remain unclear.

In our study, DK16873 and GS16568 HPAIVs isolated in 2016 in southern China possessed multiple basic amino acid residues (RERRRKR/GLF) at cleavage site of HA, which indicated that both of them were highly pathogenic AIVs (Supplementary Table S2). Animal experiment results show that both the two H5N6 viruses were able to replicate in multi-organs of inoculated ducks. DK16873 virus could cause mild infections and killed 2/5 of inoculated ducks, GS16568 virus did not cause obvious clinical symptoms, or killed inoculated ducks. Thus, our results demonstrated that the two H5N6 HPAIVs had caused different mortality in ducks. But, the results of Fisher’s exact tests showed that the mortality rate of these inoculated ducks caused by the two viruses was not significantly different.

We have further sequenced the whole genomes of the two viruses and analyzed the potential virulence determinants in the genomes of the two viruses (Supplementary Table S2). Results showed that amino acid residues Q226 and G228 (H3 influenza numbering) were observed in HA of both the two viruses, which suggested that the two viruses were prefer to bind to the avian-like receptors (Supplementary Table S2; Stevens et al., 2006). A recognized stalk deletion (58–68 amino acid residues; N6 numbering) in NA was observed in the GS16568 and DK16873 viruses, which may enhance the viral virulence toward mammals (Matsuoka et al., 2009). Both the PB2 of two viruses did not carry mutations related to mammalian adaptation (e.g., E627K and D701N) (Gabriel et al., 2013). Mutations (N30D and T215A) were observed in the M1 of two viruses, which may enhance the viral virulence in mice (Fan et al., 2009). Both the two viruses carried D92E mutation in NS1, which may increase the viral virulence of the two viruses in mice and pigs (Seo et al., 2002). In addition, 47 amino acid residues differences were observed between GS16568 and DK16873 HPAIVs, which may also contribute to the pathogenicity of the two viruses to ducks (Supplementary Table S3). Recent studies have reported that clade 2.3.4.4 H5N6 virus HE144 possessed 11 substitutions (221T and V495L in PB2; N213S, S361I, K386R, K391R and L753F in PB1; S388G and K603R in PA; and A96P and N203D in HA) was highly pathogenic to ducks (Uchida et al., 2019). Among those substitutions, V495L in PB2, N213S, K386R, and K391R in PB1 were also found between the GS16568 and DK16873 viruses (Supplementary Table S3). As described in that literature, V495L in PB2 and N213S in PB1 are part of the nuclear localization signal; and K386R and K391R in PB1 are in the cRNA-binding domain (Uchida et al., 2019). Therefore, these four substitutions between GS16568 and DK16873 HPAIVs may have influenced the function of the polymerase complex, and may associated with the different clinical signs between the GS16568 and DK16873 HPAIVs infected ducks.

As a natural reservoir of AIVs, ducks play an important role in the transmission of H5N1 from aquatic birds to terrestrial poultry (Smith et al., 2006). Ducks often contaminate water by releasing AIVs through their feces, causing other birds in the area to be infected with avian influenza. This situation is of great concern to the transmission of AIV (Kim et al., 2009). Reports have shown that 2.3.4 clade H5N5 virus isolated in 2008 in southern China caused a 25% mortality rate in contact ducks (Liu et al., 2013). Recent studies have also demonstrated that clade 2.3.4.4 H5N2, H5N6, and H5N8 viruses isolated from eastern China in 2014 could be transmitted via direct contact but were unable to kill contact ducks (Sun et al., 2016). In our study, DK16873 virus killed 2/5 of contact ducks and GS16568 virus killed 1/5 of contact duck. The results of Fisher’s exact tests showed that the mortality rate of these contact ducks caused by the two viruses was not significantly different. Both GS16568 and DK16873 viruses were shed from the respiratory tract and the digestive tract of the inoculated ducks and contact ducks at least 9 DPI, which indicated that the two viruses could be efficiently transmitted via direct contact. Our results demonstrated that clade 2.3.4.4 H5N6 viruses isolated from southern China could be transmitted via direct contact between ducks.

Previous studies have shown that the pathogenicity of AIV is associated with the host immune response (Burggraaf et al., 2014). However, there is little knowledge about the immune response of ducks infected with H5N6 virus. In this study, we quantified the expression of immune-related genes in the lungs of ducks infected with H5N6 viruses of different pathogenicity.

After influenza virus infection, host PRRs detect viral RNA to trigger a series of antiviral signaling pathways, which leads to the production of ISGs and pro-inflammatory cytokines (Wang et al., 2007). Our previous studies have shown that the expression of TLR3 and RIG-I were increased in the brains of ducks infected with H5N1 HPAIV (Wei et al., 2013). In addition, the expression of TLR3 was also significantly upregulated in the lungs and brains of H5N6 HPAIVs inoculated chickens (Gao et al., 2017). In our study, TLR7, TLR3, and MDA5 were also significantly increased in the lung of ducks infected with GS16568 and DK16873 viruses at 3 DPI. Studies have reported that RIG-I is the most important retinoic acid-inducible gene I (RIG-I)-like receptors (RLRs) for recognizing influenza virus (Kato et al., 2006). RIG-I is absent in chickens but present in ducks, which may explain why influenza viruses are more pathogenic to chickens than ducks (Barber et al., 2010). Previous studies have indicated that the expression of RIG-I was highly upregulated in response to the H5N1 AIVs in ducks (Fleming-Canepa et al., 2019). In our study, RIG-I was significantly increased in response to GS16568 and DK16873 viruses at 12 HPI and 3 DPI. Interestingly, ducks infected with DK16873 exhibited much higher expression of RIG-I than ducks infected with GS16568 at both 12 HPI and 3 DPI. Therefore, our results showed that TLR7, TLR3, RIG-I, and MDA5 were involved in the immune responses of ducks infected with waterfowl-origin H5N6 viruses, though with different expression patterns.

As noted above, viral infection activates PRRs and leads to the transcription of ISGs (e.g., Mx and OAS) and pro-inflammatory cytokines (e.g., IL-6 and IL-8). Previous studies have indicated that IFN-α and IL-6 were upregulated after infection with H11N9 LPAI virus in duck peripheral blood mononuclear cells (PBMCs) (Adams et al., 2009). Other investigations have shown that the expression of IFN-β was increased in the lungs of ducks infected with H5N1 virus (Fleming-Canepa et al., 2019). Reports showed that a higher expression of IL-6 was correlated with high replication of H9N2 virus and damage of the tested tissues (Xing et al., 2008). Our previous studies have shown that ducks infected with H5N1 HPAIVs induced high expression of IL-6 and IL-8 in brains, which may lead to the destruction of host immune responses (Wei et al., 2013). Our present results also revealed that the expression of IL-6 was significantly increased after birds were infected with the two H5N6 HPAIVs at 12 HPI and 3 DPI. Notably, ducks infected with DK16873 H5N6 virus exhibited much higher expression of IL-6 in their lungs than birds infected with GS16568 H5N6 virus. And the expression of IFN-α, IFN-β, Mx, and IL-8 were also increased at 3 DPI in the lungs of the infected ducks. Thus, our results indicated that IFN-α, IFN-β, Mx, IL-6, and IL-8 were involved in the duck immune response to H5N6 viruses.

The host innate immune system control virus replication at the early phase of infection; adaptive immunity is required to eliminate viruses in later stages of infection (Akira et al., 2006). MHC molecules are crucial for adaptive immunity: they are responsible for presenting processed antigens to activate T cells to clear the virus. Previous studies have shown that the expression of MHC-I was upregulated in ducks infected with H5N1 HPAI (Cagle et al., 2011). Studies have also indicated that MHC-I was upregulated in duck PBMC after infection with H11N9 LPAI virus (Adams et al., 2009). Our results revealed that MHC-I was significantly increased at 3 DPI in the lung of ducks infected with GS16568 and DK16873 viruses. Therefore, our results demonstrated that MHC-I was also involved in the immune response of ducks infected with H5N6 viruses.

Though our results showed that immune-related genes (e.g., IFNs and ISGs) were involved in the immune response of ducks infected with H5N6 HPAIVs, we only quantified the expression of these genes in mRNA level because we could not obtain related commercial duck antibodies of these proteins. Further studies should focus on the protein expression of these genes to clearly understand the immune response of ducks infected with the new H5N6 HPAIVs.

In conclusion, we found that the GS16568 and DK16873 viruses isolated from southern China in 2016 were able to replicate systematically in ducks and be transmitted efficiently in these birds. DK16873 virus could cause illness in infected ducks, while GS16568 virus could not lead to clinical illness. Furthermore, ducks infected with the two H5N6 viruses exhibited different expressions of immune-related genes in their lungs. Notably, the expression of RIG-I and IL-6 in response to DK16873 virus was significantly higher than for GS16568 virus at 12 HPI and 3 DPI, which may correlate with the different clinical signs in the two viruses infected ducks. Therefore, our results have provided useful information about the pathogenicity and immune response of ducks infected with the new H5N6 HPAIVs.

## Data Availability

The raw data supporting the conclusions of this manuscript will be made available by the authors, without undue reservation, to any qualified researcher.

## Author Contributions

SW and PJ designed the study, performed the experiments, and drafted the manuscript. JH, ZC, WH, WL, ZL, ZH, BZ, and JZ assisted with the animal experiments. ML, PJ, and ZQ participated in the writing of the discussion. All authors read and approved the final manuscript.

## Conflict of Interest Statement

The authors declare that the research was conducted in the absence of any commercial or financial relationships that could be construed as a potential conflict of interest.
