# Supplementary material for: Immune-Related Gene Expression in Ducks Infected With Waterfowl-Origin H5N6 Highly Pathogenic Avian Influenza Viruses
Source: Front Microbiol. 2019 Aug 2;10:1782. doi: 10.3389/fmicb.2019.01782 (PMC6687855; doi:10.3389/fmicb.2019.01782)
Supplement: Supplementary file 1 [file Table_1.docx]

**Table S1 Replication of H5N6 avian influenza viruses in the tissues of dead contact ducks**

| **Strains** | **Dead time** | **Virus replication in organs (log_10_EID_50_/100 mg) ^a^** | | | | | | |
| --- | --- | --- | --- | --- | --- | --- | --- | --- |
|  |  | **Liver** | **Spleen** | **Lung** | **Kidney** | **Intestine** | **Pancreas** | **Bursa of Fabricius** |
| GS16568-contact duck | 4 DPI | 4.5 | 4.25 | 6.5 | 5.5 | 3 | 2.75 | 3.5 |
| DK16873-contact duck | 5 DPI | 6.25 | 2.5 | 6.25 | 5.5 | 4.5 | 2.25 | 3.5 |
| DK16873-contact duck | 6 DPI | 5.75 | 4.25 | 5.5 | 5.5 | 4.5 | 2.25 | 5.25 |

a: For statistical analysis, a value of 1.5 was assigned if the virus was not detected from the undiluted sample in three embryonated hen eggs.

Viral titers were expressed as mean ± SD in log_10_EID_50_/100 mg of tissues
