# Supplementary material for: Immune-Related Gene Expression in Ducks Infected With Waterfowl-Origin H5N6 Highly Pathogenic Avian Influenza Viruses
Source: Front Microbiol. 2019 Aug 2;10:1782. doi: 10.3389/fmicb.2019.01782 (PMC6687855; doi:10.3389/fmicb.2019.01782)
Supplement: Supplementary file 2 [file Table_2.docx]

| **Viral proteins** | **Phenotypic effect** | **Mutations or molecular feature** | **GS16568** | **DK16873** |
| --- | --- | --- | --- | --- |
| HA | Cleavage site  Characteristic of HPAIVs | PLRERRRKR/GLF | Yes | Yes |
|  | Altered receptor binding specificity  (H3 numbering) ^a^ | Q226L | Q | Q |
|  |  | G228S | G | G |
| NA | Increased virulence in mice  (N6 numbering) ^b^ | Stalk deletion | Yes | Yes |
| PB2 | Mammalian adaptation | E627K | E | E |
|  |  | D701N | D | D |
| M1 | Increased virulence in mice | N30D | D | D |
|  |  | T215A | A | A |
| NS1 | Increased virulence in mice | D92E | E | E |

**Table S2 Molecular analysis of important amino acid residues in the genome of the two H5N6 HPAIVs**

a: The H3 numbering system is used.

b: The N6 numbering system is used.
