# Supplementary material for: Immune-Related Gene Expression in Ducks Infected With Waterfowl-Origin H5N6 Highly Pathogenic Avian Influenza Viruses
Source: Front Microbiol. 2019 Aug 2;10:1782. doi: 10.3389/fmicb.2019.01782 (PMC6687855; doi:10.3389/fmicb.2019.01782)
Supplement: Supplementary file 3 [file Table_3.docx]

**Table S3 Summary of amino acid residues differences between GS16568 and DK16873 HPAIVs**

| **PB2** | **152^a^** | **178** | **292** | **398** | **441** | **448** | **456** | **495** | **559** | **588** |  |  |  |  |  |
| --- | --- | --- | --- | --- | --- | --- | --- | --- | --- | --- | --- | --- | --- | --- | --- |
| GS16568 | T | A | V | I | N | N | S | V | S | V |  |  |  |  |  |
| DK16873 | A | T | I | L | D | T | N | L | N | I |  |  |  |  |  |
| **PB1** | **12** | **75** | **195** | **213** | **298** | **311** | **336** | **386** | **391** | **398** | **414** | **515** | **576** | **667** | **746** |
| GS16568 | I | G | M | N | V | E | V | K | K | D | V | P | P | I | T |
| DK16873 | V | E | I | S | L | V | A | R | R | E | M | S | L | V | I |
| **PA** | **55** | **351** |  |  |  |  |  |  |  |  |  |  |  |  |  |
| GS16568 | D | G |  |  |  |  |  |  |  |  |  |  |  |  |  |
| DK16873 | N | E |  |  |  |  |  |  |  |  |  |  |  |  |  |
| **HA** | **51** | **56** | **146** | **337** |  |  |  |  |  |  |  |  |  |  |  |
| GS16568 | R | K | V | L |  |  |  |  |  |  |  |  |  |  |  |
| DK16873 | K | R | M | S |  |  |  |  |  |  |  |  |  |  |  |
| **NP** | **34** | **112** | **125** |  |  |  |  |  |  |  |  |  |  |  |  |
| GS16568 | G | D | S |  |  |  |  |  |  |  |  |  |  |  |  |
| DK16873 | S | N | N |  |  |  |  |  |  |  |  |  |  |  |  |
| **NA** | **50** | **59** | **75** | **251** | **310** | **331** | **430** |  |  |  |  |  |  |  |  |
| GS16568 | I | N | K | K | K | T | T |  |  |  |  |  |  |  |  |
| DK16873 | M | K | N | R | R | I | A |  |  |  |  |  |  |  |  |
| **M1** | - | - | - | - | - | - | - | - | - | - | - | - | - | - | - |
| GS16568 | - | - | - | - | - | - | - | - | - | - | - | - | - | - | - |
| DK16873 | - | - | - | - | - | - | - | - | - | - | - | - | - | - | - |
| **M2** | **10** | **13** |  |  |  |  |  |  |  |  |  |  |  |  |  |
| GS16568 | P | H |  |  |  |  |  |  |  |  |  |  |  |  |  |
| DK16873 | S | N |  |  |  |  |  |  |  |  |  |  |  |  |  |
| **NS1** | **134** | **147** | **221** | **225** |  |  |  |  |  |  |  |  |  |  |  |
| GS16568 | N | E | I | I |  |  |  |  |  |  |  |  |  |  |  |
| DK16873 | D | K | V | V |  |  |  |  |  |  |  |  |  |  |  |

a：The numbers represent the position of Amino-acid residues in viral proteins.
